# CNS Non-Hodgkin's Lymphoma With Atypical T2 Hyperintensity and Necrosis Mimicking Glioblastoma Multiforme: A Radiological Enigma

**DOI:** 10.7759/cureus.89353

**Published:** 2025-08-04

**Authors:** Mohammed Haroon Ahmed, Mukarram Mohammed Abdul, Ayman Nadeem, Nigama Yaratapally, Mirza Abid, Abdul Malik Mohammad, Mohammed Abdul Muhaimin Ali, Krishna S Athmakuri, Omer Farooq Mohammed, Tushar Bhadra

**Affiliations:** 1 Radiodiagnosis, Gandhi Medical College, Hyderabad, IND; 2 General Surgery, Osmania Medical College, Hyderabad, IND; 3 Medicine, Osmania Medical College, Hyderabad, IND; 4 Medicine, Gandhi Medical College, Hyderabad, IND; 5 Internal Medicine, Osmania Medical College, Hyderabad, IND; 6 Surgery, Dhaka Medical College and Hospital, Dhaka, BGD

**Keywords:** atypical mri, brain tumor, case report, cns lymphoma, glioblastoma mimic, histopathology, neuroradiology, t2 hyperintensity

## Abstract

A 45-year-old female presented with a 15-day history of headache and blurred vision. MRI of the brain revealed multiple irregular, T2-hyperintense lesions with significant surrounding edema, central necrosis, peripheral rim enhancement, and corpus callosum involvement resulting in a "butterfly" appearance. These imaging features led to an initial radiological impression of multifocal glioblastoma multiforme. Subsequent histopathological examination of a brain biopsy provided a definitive diagnosis of CNS non-Hodgkin's lymphoma (CNS-NHL). The biopsy showed dense perivascular cuffing by atypical large lymphocytes with notable mitoses and adjacent edema. This report illustrates an imaging presentation of CNS lymphoma that differed from common patterns, particularly the prominent T2 hyperintensity within the enhancing rim and central necrosis, posing a diagnostic challenge resolved using histopathological analysis. The findings emphasize the importance of tissue diagnosis in cases with complex neuroimaging presentations suggestive of high-grade glioma.

## Introduction

Primary CNS non-Hodgkin's lymphoma (CNS-NHL) is a rare and aggressive extranodal tumor confined to the brain, eyes, spinal cord, or leptomeninges, with no evidence of systemic involvement at diagnosis [1]. It accounts for 2-3% of all primary brain tumors and less than 1% of all NHL cases [2,3]. While its incidence is on the rise, particularly in the elderly, the prognosis remains one of the poorest among all NHLs. Radiologically, CNS-NHL in immunocompetent patients typically presents as a solitary, homogeneously enhancing lesion located in the periventricular white matter [2]. However, atypical presentations, including multifocality, ring enhancement, and central necrosis, can occur, making it difficult to distinguish from other intracranial pathologies, most notably glioblastoma multiforme (GBM) [4]. GBM, the most common primary malignant brain tumor in adults, frequently presents with ring enhancement, central necrosis, and can also cross the corpus callosum, creating a "butterfly" pattern similar to that seen in some cases of CNS-NHL [5].

## Case presentation

A 45-year-old female presented to the emergency department with a 15-day history of continuous, gradually progressive headaches associated with blurring of vision. She also reported a one-week history of non-bilious vomiting. Her past medical history was significant for type 2 diabetes mellitus, for which she was on irregular medication. She noted significant weight loss, easy fatigability, and symptoms of polyuria and polydipsia.

On examination, the patient was conscious and coherent with a Glasgow Coma Scale (GCS) score of 15/15. Her vital signs were stable. A comprehensive neurological examination, including cranial nerves, motor, sensory, and cerebellar functions, was unremarkable. Although the patient’s poorly controlled diabetes could have contributed to relative immune dysfunction, she had no history of opportunistic infections or other conditions suggestive of profound immunosuppression (e.g., HIV infection or post-transplant status). Therefore, she was considered an immunocompetent host for the classification of CNS-NHL. Based on the patient's level of independence and symptom burden, her preoperative Karnofsky Performance Status (KPS) was estimated to be 80. Laboratory investigations were within normal limits, except for an elevated random blood glucose of 280 mg/dL and an HbA1C of 9.5%, as shown in Table 1.

**Table 1:** Summary of patient’s laboratory workup ALP: alkaline phosphatase; HCT: hematocrit; MCV: mean corpuscular volume; SGOT: serum glutamic oxaloacetic transaminase; SGPT: serum glutamic pyruvic transaminase; WBC: white blood cells

Parameter	Patient value	Reference range
Haemoglobin (g/dL)	12.1	12.0–15.5
WBC (cells/mm^3^)	8,930	4,000–11,000
Platelets (/mm^3^)	243,000	150,000–450,000
MCV (fL)	78.6	80–100
HCT (%)	35.2	36–46
Blood urea (mg/dL)	31	7–20
Serum creatinine (mg/dL)	0.39	0.6–1.1
Serum total bilirubin (mg/dL)	0.33	0.3–1.2
Serum direct bilirubin (mg/dL)	0.07	0–0.3
SGOT (U/L)	7	5–40
SGPT (U/L)	10	7–56
ALP (U/L)	44	44–147
Albumin (g/dL)	3.1	3.5–5.2
Globulin (g/dL)	3	2.0–3.5
Total protein (g/dL)	5.62	6–8.3
Serum Ca2+ (mg/dL)	8.2	8.5–10.5
Serum PO4 3- (mg/dL)	2.65	2.5–4.5
Serum Na+ (mEq/L)	136	135–145
Serum K+ (mEq/L)	3.9	3.5–5.0
Serum Cl- (mEq/L)	94	98–106
Uric acid (mg/dL)	1.95	2.4–6.0

**Figure 1:**
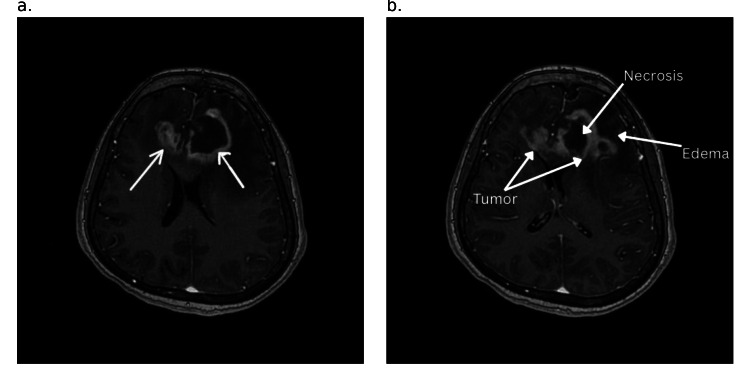
Axial contrast-enhanced MRI image The image shows a peripherally enhancing tumor (shown in white arrow in figure 1a) in the left anterior frontal lobe extending into the right frontal lobe through the genu of the corpus callosum: a. At the level of the body of the lateral ventricles. b. At the level of the capsuloganglionic region MRI: magnetic resonance imaging

**Figure 2:**
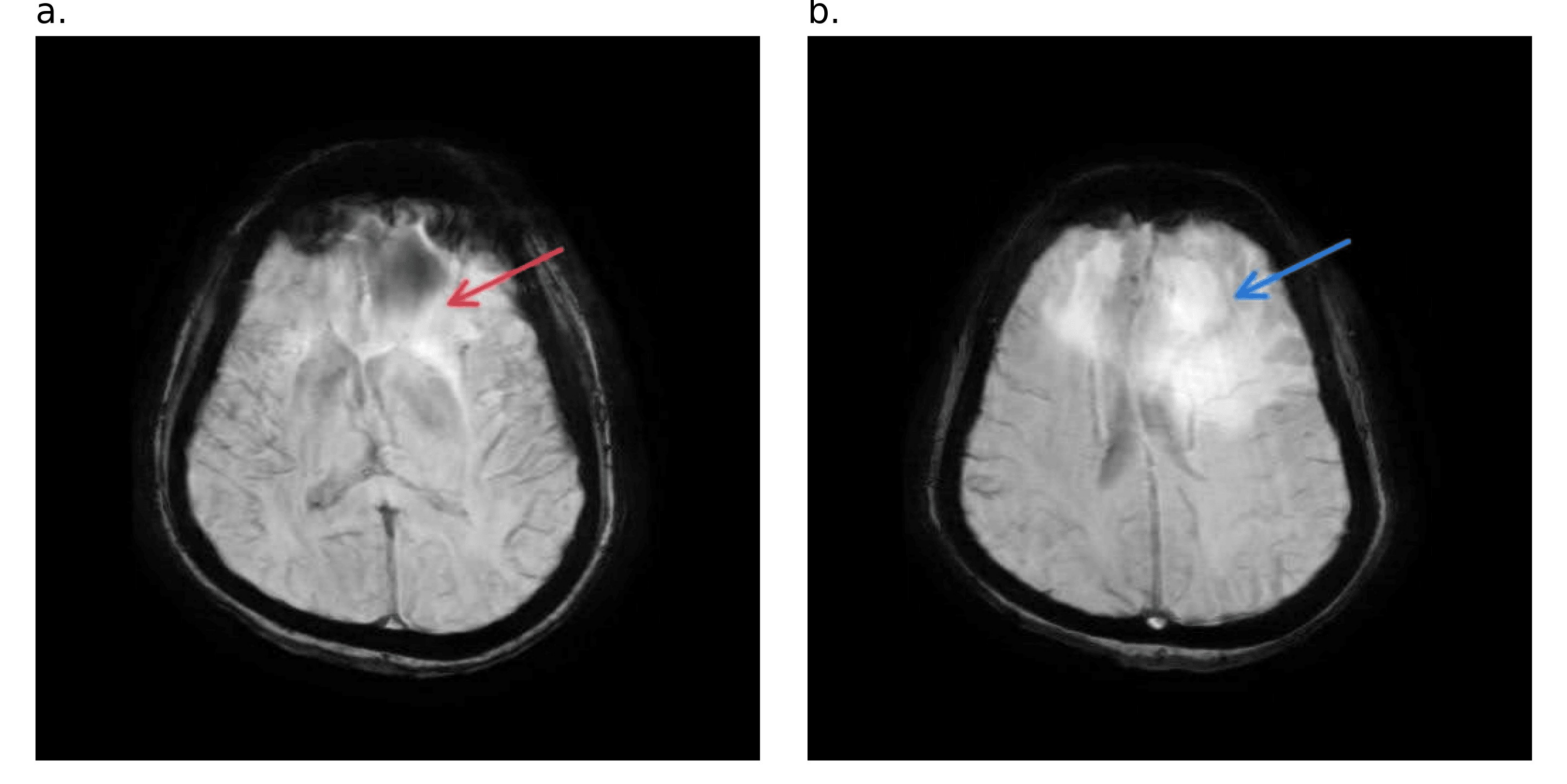
Axial SWI MRI image a. At the level of the capsuloganglionic region. b. At the level of the body of the lateral ventricle MRI: magnetic resonance imaging; SWI: susceptibility weighted imaging

**Figure 3:**
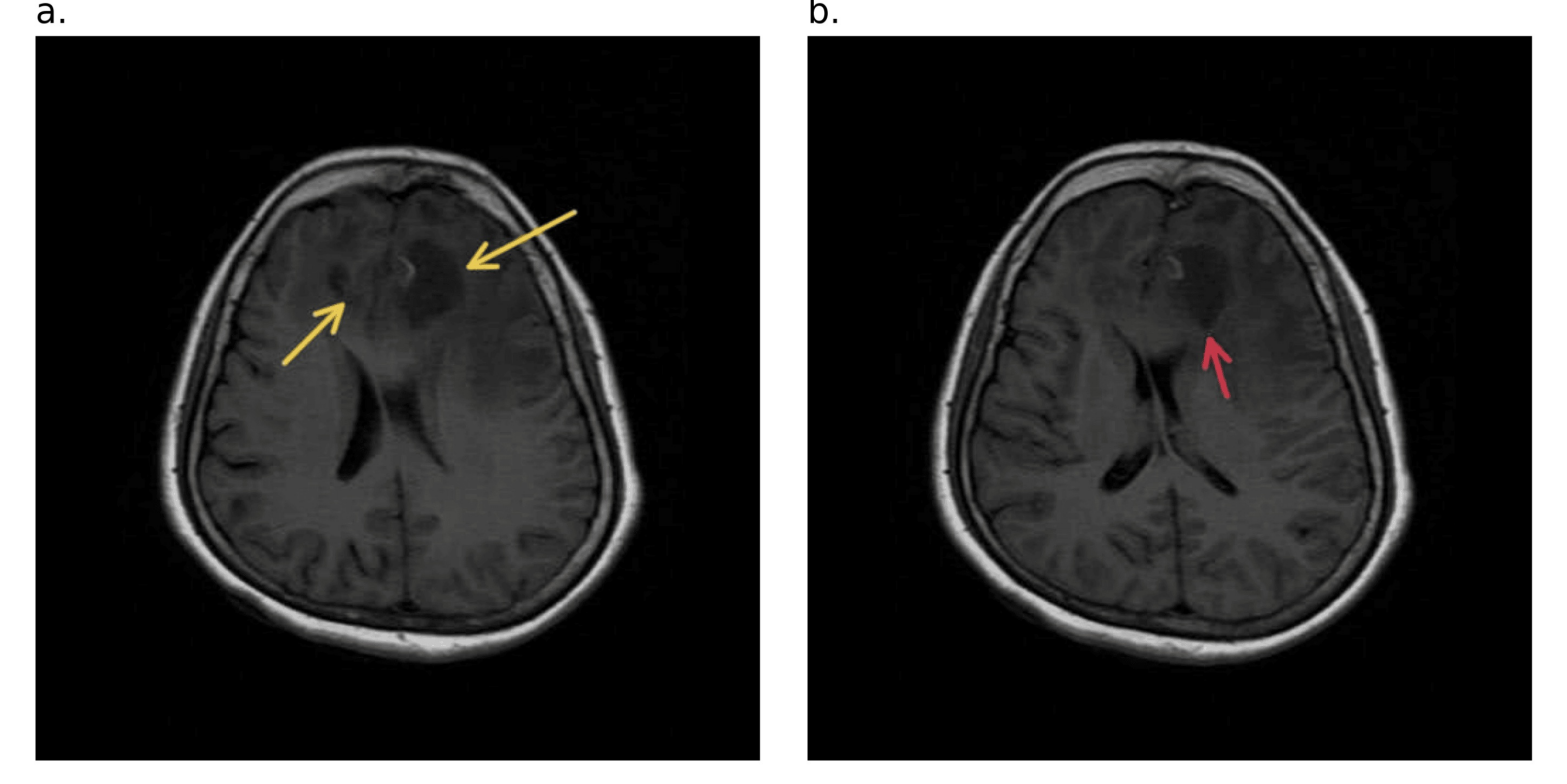
Axial T1-weighted MRI image a. The images show the peripherally enhancing tumor (yellow arrows) in the left frontal lobe at the level of the body of the lateral ventricles, and b. (red arrow) at the level of the capsuloganglionic region MRI: magnetic resonance imaging

**Figure 4:**
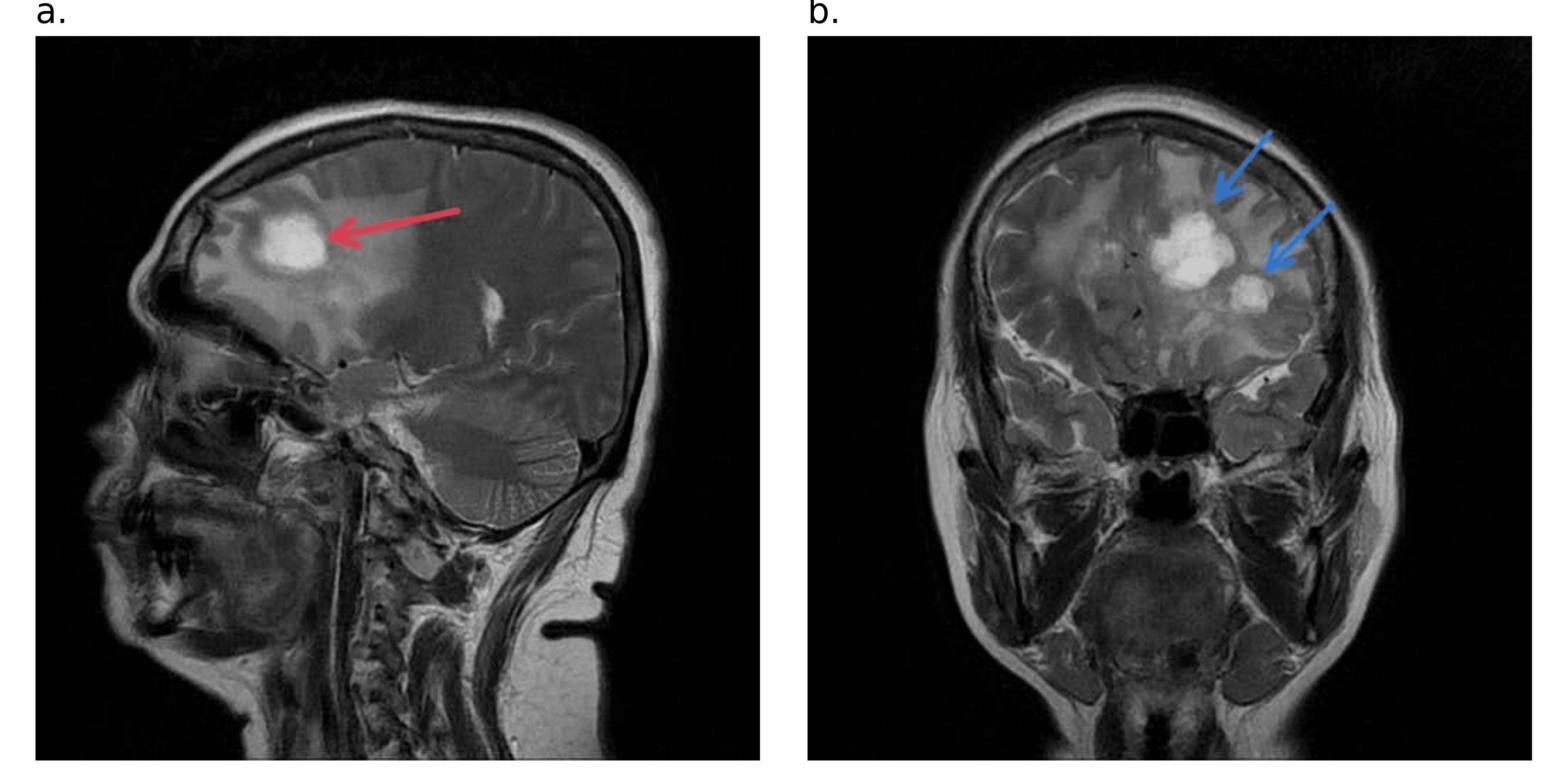
T2-weighted MRI image a. Sagittal view showing tumor (red arrow) in the anterior lobe. b. Coronal view showing multiple tumors (blue arrows) within the left anterior frontal lobe MRI: magnetic resonance imaging

**Figure 5:**
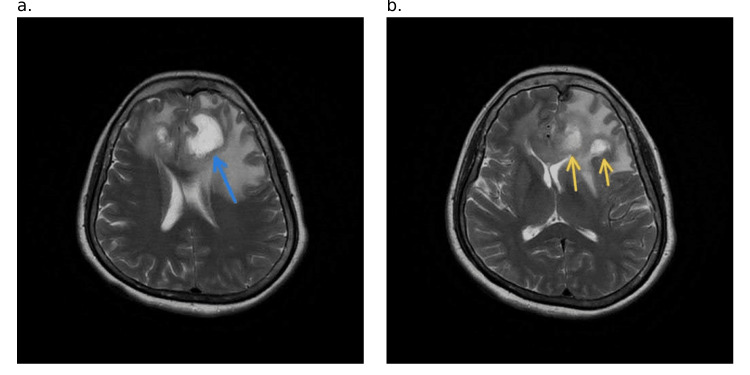
Axial T2-weighted MRI image a. Peripherally enhanced tumor (blue arrow) extending into the right frontal lobe at the level of the body of the lateral ventricles. b. Multiple peripherally enhancing tumors (yellow arrows) at the level of the capsuloganglionic region MRI: magnetic resonance imaging

**Figure 6:**
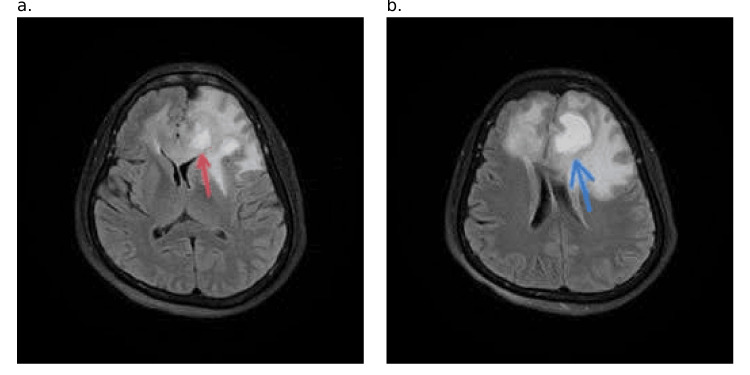
Axial FLAIR MRI image a. Peripherally enhancing tumor (red arrow) at the level of the capsuloganglionic region, and b. (blue arrow) at the level of the body of the lateral ventricles FLAIR: fluid-attenuated inversion recovery; MRI: magnetic resonance imaging

**Figure 7:**
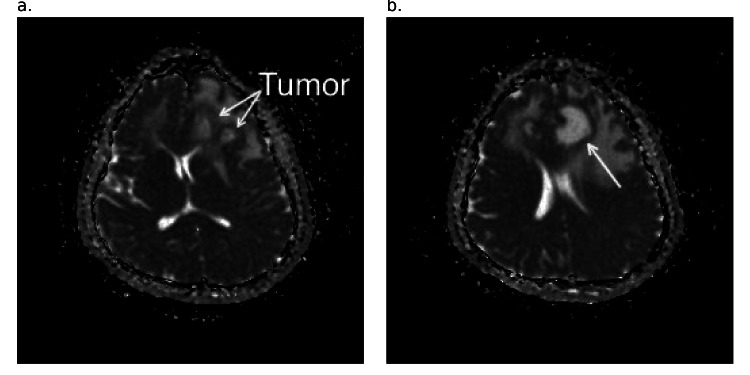
Axial ADC map MRI image a. At the level of the capsuloganglionic region b. At the level of the body of the lateral ventricles, a peripherally enhancing tumor(white arrow) ADC: apparent diffusion coefficient; MRI: magnetic resonance imaging

No corticosteroids were administered before the diagnostic workup. Given the clinical suspicion of an intracranial space-occupying lesion (ICSOL), a contrast-enhanced MRI of the brain was performed on June 5, 2024, which revealed multiple irregular, oval-shaped lesions with features suggestive of a high-grade neoplasm. The largest lesion, measuring approximately 3.2 x 2.2 x 2.8 cm³, was located in the left frontal lobe. Smaller lesions were noted in the left parietal and right frontal lobes. The lesions were T1-hypointense and T2/FLAIR-hyperintense with extensive surrounding vasogenic edema. A key finding was the extension of the pathology across the genu of the corpus callosum, creating a butterfly-shaped pattern. After contrast administration, the lesions demonstrated thick, irregular, peripheral ring enhancement with central non-enhancing areas suggestive of necrosis (Figures 1-7, Table 2). These findings led to a primary radiological diagnosis of multifocal GBM (WHO Grade IV). Consequently, a preoperative management plan to proceed with surgical excision was made for a definitive histopathological diagnosis.

**Table 2:** Summary of key MRI findings ADC: apparent diffusion coefficient; FLAIR: fluid-attenuated inversion recovery; MRI: magnetic resonance imaging; NHL: non-Hodgkin's lymphoma; SWI: susceptibility weighted imaging

MRI sequence	Key findings
Contrast-enhanced MRI	Peripherally enhancing frontal lobe lesion crossing the midline
SWI	No blooming, rules out hemorrhagic component
T1-weighted	Iso -to hypotense mass with midline extension
T2 weighted	Hyperintense lesion with surrounding edema
FLAIR	Hyperintense mass with indistinct margins
ADC	Diffusion restriction indicating high cellularity (typical of NHL)

**Figure 8:**
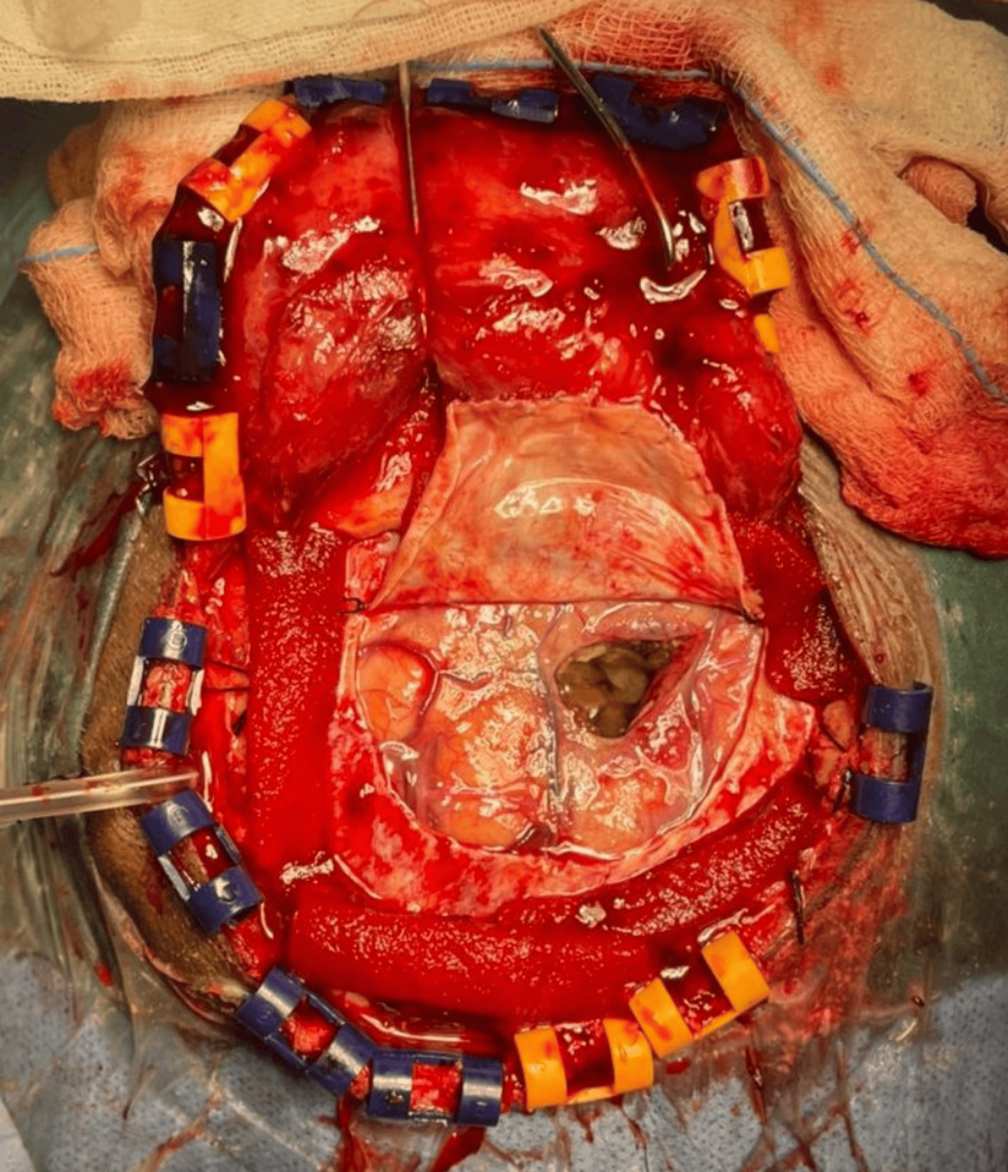
Gross intraoperative appearance of the intracranial lesion This image from the left frontal craniotomy shows the exposed mass after retraction of the scalp and dura mater

On June 10, 2024, the patient underwent a left frontal craniotomy with excision of the ICSOL (Figure 8). A 1 cc sample of cystic fluid was aspirated, and the mass was separated from the surrounding brain parenchyma. The excised specimen consisted of two gray-white to gray-brown soft tissue fragments and was sent for histopathological examination.

**Figure 9:**
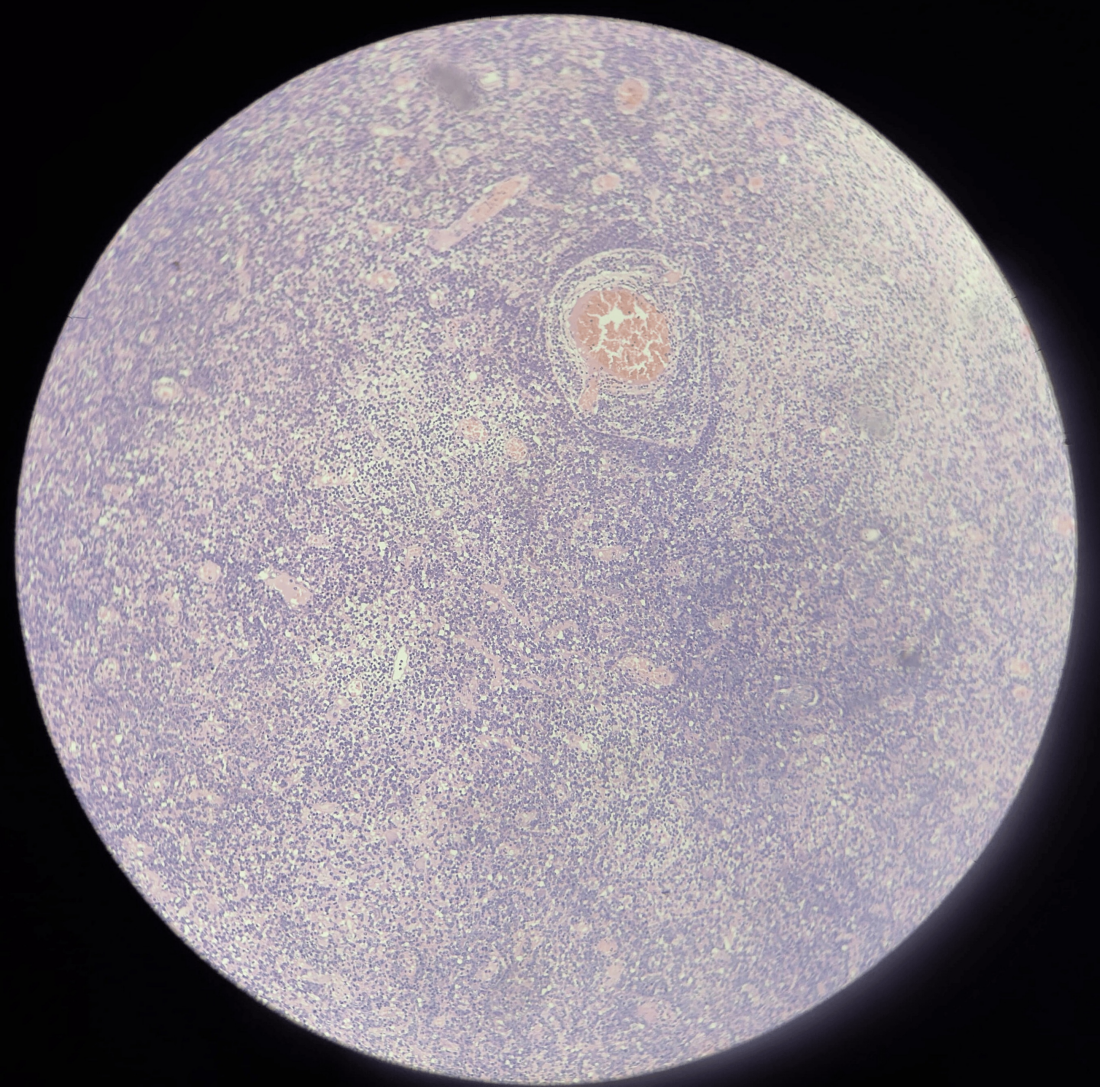
Low-power micrograph of the brain biopsy A blood vessel is seen in the center, surrounded by a dense, well-defined aggregate of malignant lymphocytes, demonstrating the characteristic feature of perivascular cuffing. The surrounding glial tissue is also diffusely infiltrated by these atypical cells

**Figure 10:**
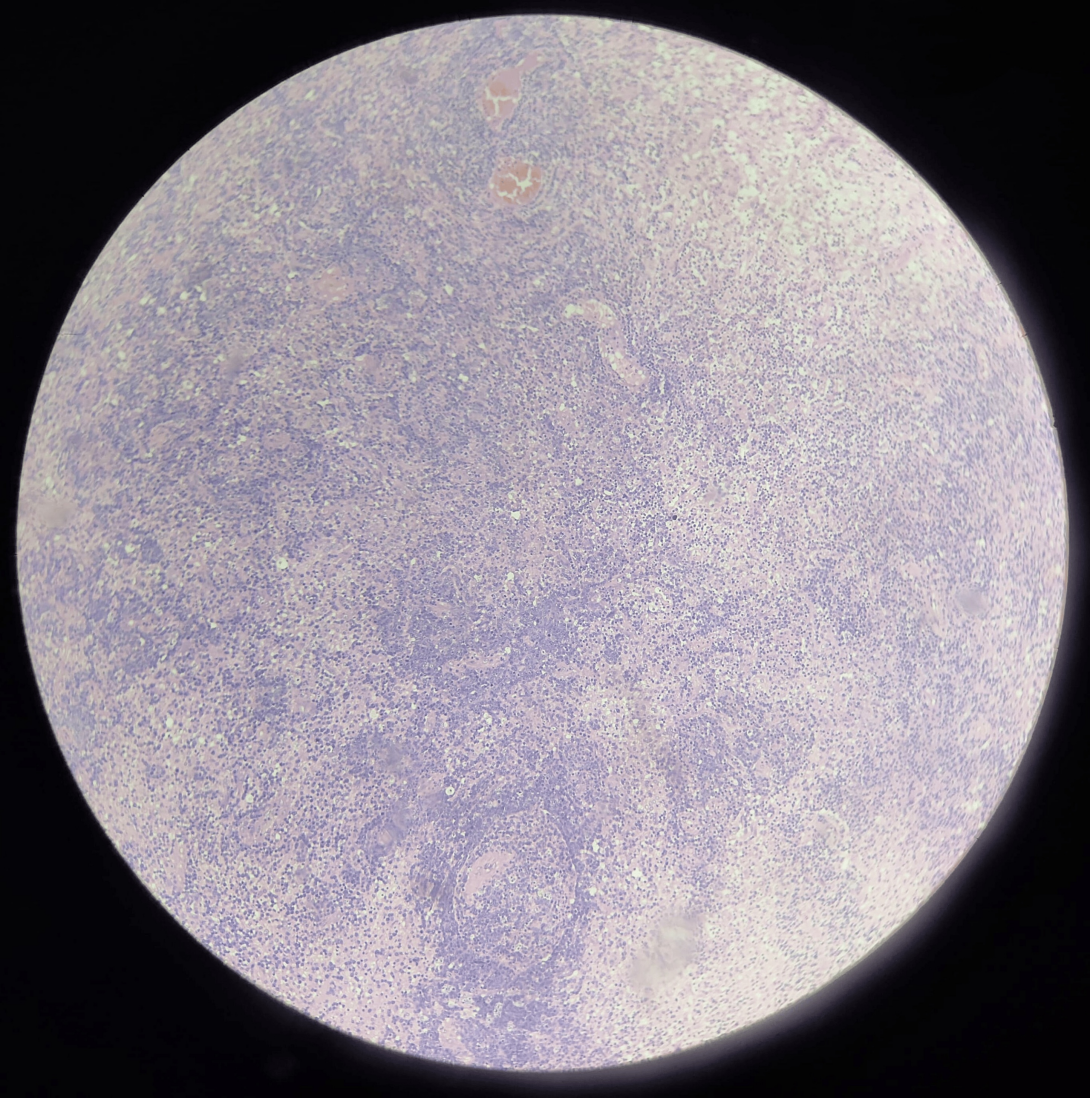
Low-power view illustrating the extensive and diffuse nature of the lesion The normal brain architecture is largely replaced by solid sheets of atypical lymphocytes. Multiple areas of angiocentric lymphoid collections are visible throughout the tissue

**Figure 11:**
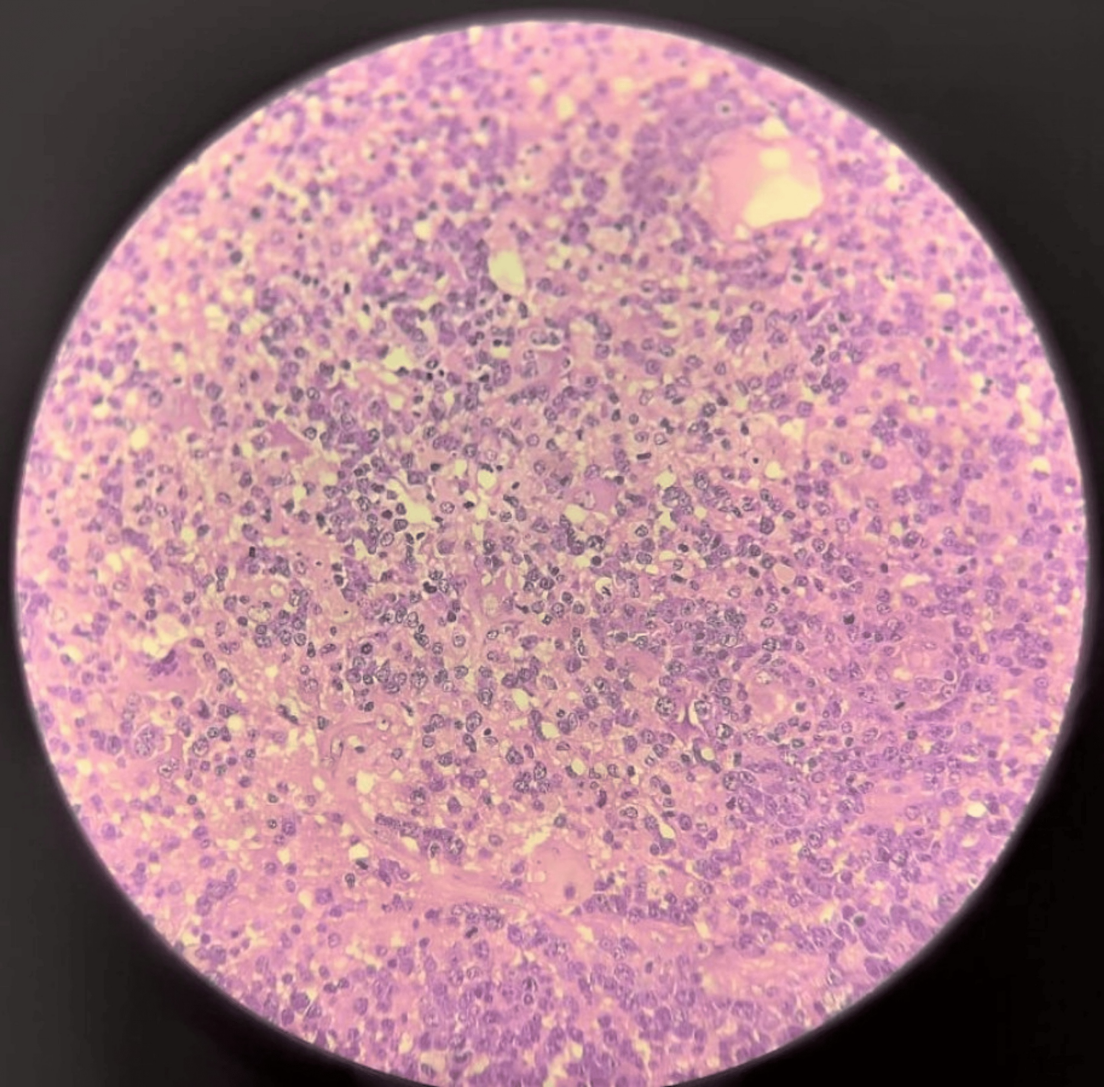
High-power view showing the cellular details of the lymphoma The infiltrate is composed of a mixed population of atypical large lymphocytes, macrophages, and few small lymphocytes. The atypical cells are large, with round nuclei and scant cytoplasm. Numerous mitotic figures are present, indicating a high rate of cell proliferation

**Figure 12:**
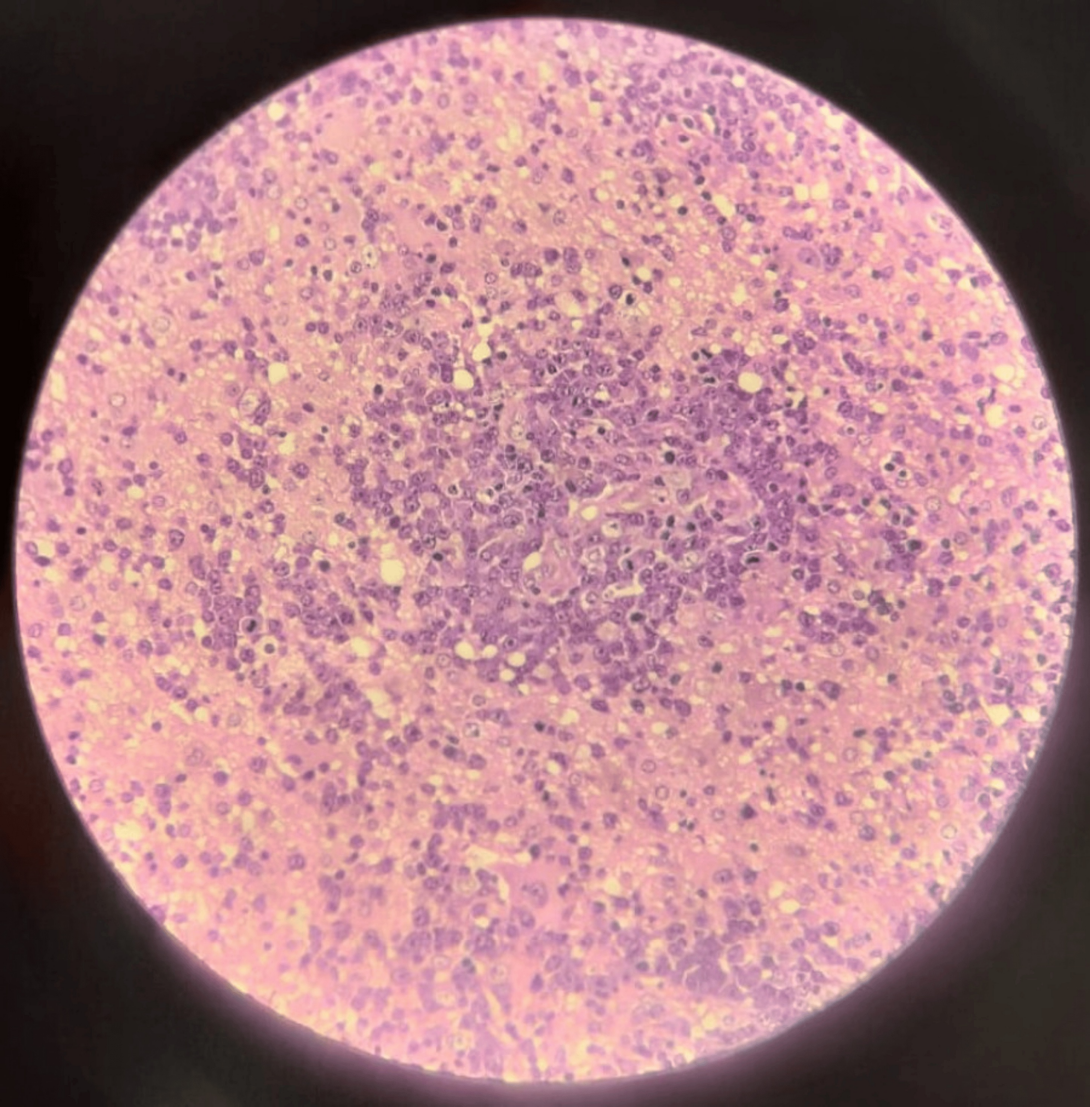
High-power magnification detailing the cytology of the malignant cells The atypical lymphocytes are large with round nuclei, visible nucleoli, and minimal cytoplasm. These cells are seen forming dense sheets, admixed with scattered smaller reactive lymphocytes and macrophages

**Figure 13:**
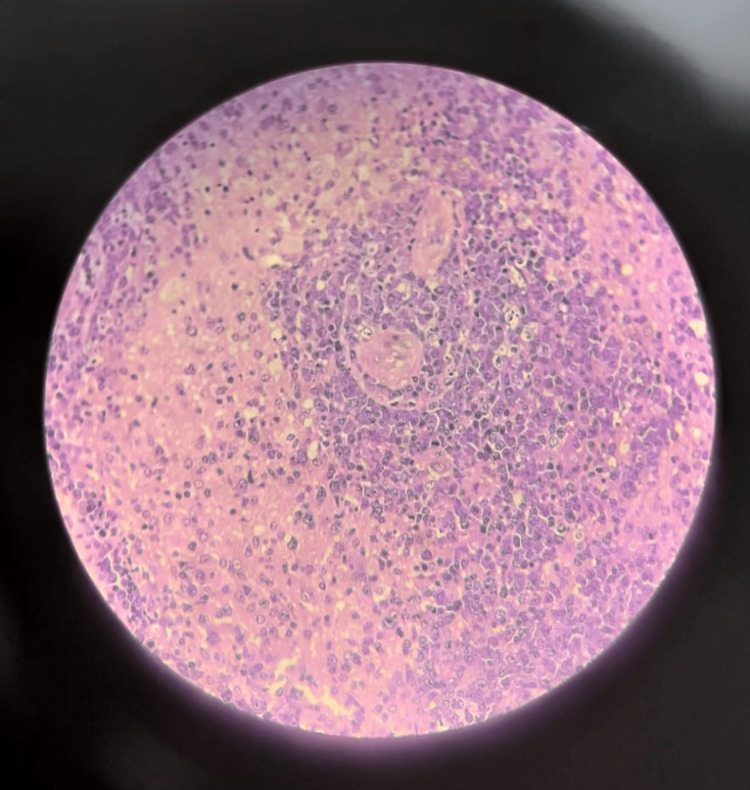
High-power view demonstrating the angioinvasive nature of the lymphoma A small blood vessel is heavily infiltrated by atypical large lymphocytes. The surrounding brain parenchyma shows edema and a dense infiltrate of these malignant cells

The histopathology report, available as of June 17, 2024, described glial tissue with dense perivascular cuffing by atypical, large lymphocytes, admixed with macrophages. These atypical lymphocytes extended into the intervening areas as solid sheets, showing large, round nuclei with prominent nucleoli, numerous mitoses, and scanty cytoplasm (Figures 9-13). These features were inconsistent with GBM and were diagnostic of CNS-NHL. Immunohistochemistry was recommended for further subtyping. The patient had an uneventful postoperative course with complete resolution of her presenting symptoms of headache and blurred vision. No new neurological deficits were noted upon discharge. At the initial follow-up, she maintained a stable functional status corresponding to a KPS of 90.

## Discussion

The diagnosis of primary CNS-NHL can be a significant challenge, particularly when its radiological features mimic those of GBM, as demonstrated in this case [6,7]. The overlapping imaging characteristics can lead to diagnostic delays, which is critical given that prompt and accurate diagnosis is essential for improving patient outcomes [8]. This case highlights the diagnostic dilemma posed by an atypical presentation of CNS-NHL and reinforces the indispensable role of histopathology in reaching a definitive diagnosis. In immunocompetent patients, CNS-NHL typically appears on MRI as a solitary, intensely and homogeneously enhancing lesion located in the periventricular white matter [9-11]. Due to its high cellularity and high nuclear-to-cytoplasmic ratio, the lesion is often hyperdense on unenhanced CT scans, shows restricted diffusion on DWI sequences, and appears iso- to hypointense on T2-weighted images [9,12,13]. In contrast, GBM, the most common primary malignant brain tumor, characteristically presents with thick, irregular ring enhancement surrounding a central area of necrosis and is associated with significant vasogenic edema [6].

Our patient presented with multifocal lesions showing peripheral ring enhancement, central necrosis, and extension across the corpus callosum to form a "butterfly" pattern. While these features are highly suggestive of GBM, they represent a known, albeit atypical, presentation of CNS-NHL [6,9]. Studies have reported that ring enhancement can be seen in 10-15% of CNS-NHL cases in immunocompetent patients, and multifocality is observed in up to 40% of cases [10,12]. The presence of these atypical features makes the radiological differentiation from GBM exceptionally difficult based on conventional imaging alone [6,13]. Typically, CNS-NHL presents as a solitary, homogeneously enhancing lesion located in the periventricular white matter, and often appears iso- to hypointense on T2-weighted images with minimal necrosis and surrounding edema [9,10,11]. In contrast, our patient exhibited multifocal lesions with irregular peripheral ring enhancement, central necrosis, and marked T2 hyperintensity: features more commonly associated with glioblastoma [6,13,14]. This atypical imaging profile significantly contributed to the initial diagnostic confusion.

Although advanced imaging techniques can provide additional clues, significant overlap remains. For example, CNS-NHL is characterized by markedly restricted diffusion (low ADC values) due to its dense cellularity, which is typically lower than that of high-grade gliomas [12,14]. While our patient's MRI did show diffusion restriction consistent with lymphoma, the other features strongly favored glioblastoma, underscoring the limitations of relying solely on imaging for diagnosis [15]. This ambiguity necessitates a tissue diagnosis, as treatment strategies for the two malignancies differ fundamentally [7].

Ultimately, the definitive diagnosis in this case was established by histopathological examination. The finding of dense perivascular cuffing by atypical large lymphocytes is a pathognomonic feature of CNS-NHL [16-18]. This histological pattern, where tumor cells accumulate around blood vessels, was the crucial finding that differentiated this tumor from a glioma. This underscores that despite advances in neuroimaging, brain biopsy remains the gold standard for diagnosis, especially when imaging findings are equivocal or atypical [6,13]. The distinction is vital, as CNS-NHL is primarily treated with high-dose methotrexate-based chemotherapy, whereas the standard of care for GBM involves surgical resection followed by radiation and temozolomide [7]. An initial misdiagnosis could lead to inappropriate and ineffective treatment.

## Conclusions

CNS-NHL can radiologically mimic GBM, particularly when presenting with atypical features such as significant central necrosis and T2 hyperintensity, alongside corpus callosum involvement. This report highlights the diagnostic challenge posed by such overlapping imaging features and reinforces the indispensable role of early histopathological confirmation. Timely and accurate diagnosis is crucial as management pathways for CNS-NHL and GBM diverge significantly. Clinicians should maintain a high index of suspicion for CNS-NHL, even with GBM-like imaging, and pursue tissue diagnosis to guide optimal treatment and improve patient outcomes.
